# Case of Disarticulation of the Left Condyle of the Lower Jaw

**Published:** 1850-10

**Authors:** W. R. Beaumont

**Affiliations:** Professor of Surgery in the University of Toronto, Canada.


### ARTICLE XV.

*Case of Disarticulation of the Left Condyle of the Lower Jaw, with Excision of nearly the Left Half of the Bone, on account of a very Large Cartilaginous Tumor, Growing from, and Occupying the Site of all this Part of the Bone, save the Condyle and Neck.* By W. R. Beaumont, Professor of Surgery in the University of Toronto, Canada.

This patient, a child, aged seven, was admitted into the Toronto Hospital, September 17, 1849. The tumor, on his admission, extended upwards to the zygoma and malar bone, almost covering the temporo-maxillary articulation. It reached downwards to fully an inch below the angle of the jaw, extending inwards into the mouth as far as the mesial plane backwards, beyond the ramus of the jaw, and forwards to the posterior bicuspid. It pushed the tongue quite to the right of the mesial plane, concealed the velum, and almost completely filled the isthmus faucium. The molar teeth of the upper jaw were deeply imbedded in the tumor, which kept the mouth at all times open, with a constant dribbling of saliva, the upper and lower incisors not meeting by fully half an inch. The tumor had been first observed three months back. September 25, 1849: Professor Beaumont performed the operation for its removal, commencing by making a curved incision, (the concavity upwards,) extending from the lobule of the ear to the angle of the mouth, dissecting off the integuments from the tumor. The tumor was firmly wedged in under the malar bone. The outer wall of the jaw was cut vertically through with a small straight saw. The section was then at one stroke completed with a strong bone forceps. The condyle was disarticulated by being firmly grasped in a forceps, the joint being opened by dividing the external lateral ligament and capsule.

The patient did very well. A small stationary fistula was formed in the cheek, which eventually healed. On December 1, 1849, the patient was quite well. The right half of the lower jaw was drawn a very little towards the left side, about one-eighth of an inch. The external cicatrix was a mere line.

[*Medical Gazette.*

